# Uterine rhabdomyosarcoma complicated by cerebral venous thrombosis and uterine inversion in a young woman: case report and literature review

**DOI:** 10.1186/s12905-021-01459-2

**Published:** 2021-08-26

**Authors:** Hongfa Peng, Jingjing Jiang, Xianghua Huang

**Affiliations:** 1 Department of Obstetrics and Gynecology, Second Hospital of Hebei Medical University, Heping West Road No. 215, Shijiazhuang City, 050000 Hebei Province China; 2 Department of Obstetrics and Gynecology, Hebei General Hospital, Shijiazhuang, 050051 China

**Keywords:** Uterine rhabdomyosarcoma, Abnormal uterine bleeding, Uterine inversion, Cerebral venous thrombosis, Case report

## Abstract

**Background:**

Uterine rhabdomyosarcoma is an extremely rare malignant tumor that usually affects young women and has a poor prognosis.

**Case presentation:**

A 19-year-old nulliparous woman presented to the emergency department under sedation due to seizures. Imaging examination revealed cerebral venous thrombosis. During thrombolytic therapy, she developed vaginal bleeding followed by uterine inversion secondary to uterine rhabdomyosarcoma. The inverted uterus was mistaken for a cervical tumour and was removed vaginally. The patient’s disease progressed despite chemotherapy with vincristine, actinomycin D and cyclophosphamide and she died within 6 months. To our knowledge, this is the first case of uterine rhabdomyosarcoma complicated with cerebral venous thrombosis.

**Conclusions:**

Malignancy is an important diagnostic in patients with cerebral venous thrombosis with no obvious cause. This case demonstrates the importance of considering uterine neoplasms in the differential diagnosis of adolescent girls with abnormal uterine bleeding. Further, careful anatomical evaluation of vaginal masses should be performed prior to surgical intervention.

## Background

Rhabdomyosarcoma is extremely rare in the uterine corpus and principally affects young women [1, 2]. It is an under-recognized aggressive neoplasm [3]. Herein, we present a case of uterine rhabdomyosarcoma complicated by cerebral venous thrombosis (CVT) and uterine inversion in a young woman. A PubMed search was conducted of English articles published using the search terms uterine rhabdomyosarcoma and uterine inversion from 2005 (first reported case) until January 2021; this identified 5 cases of uterine rhabdomyosarcoma complicated with uterine inversion. To our knowledge, this is the first case of uterine rhabdomyosarcoma complicated with CVT.

## Case presentation

**Fig. 1:**
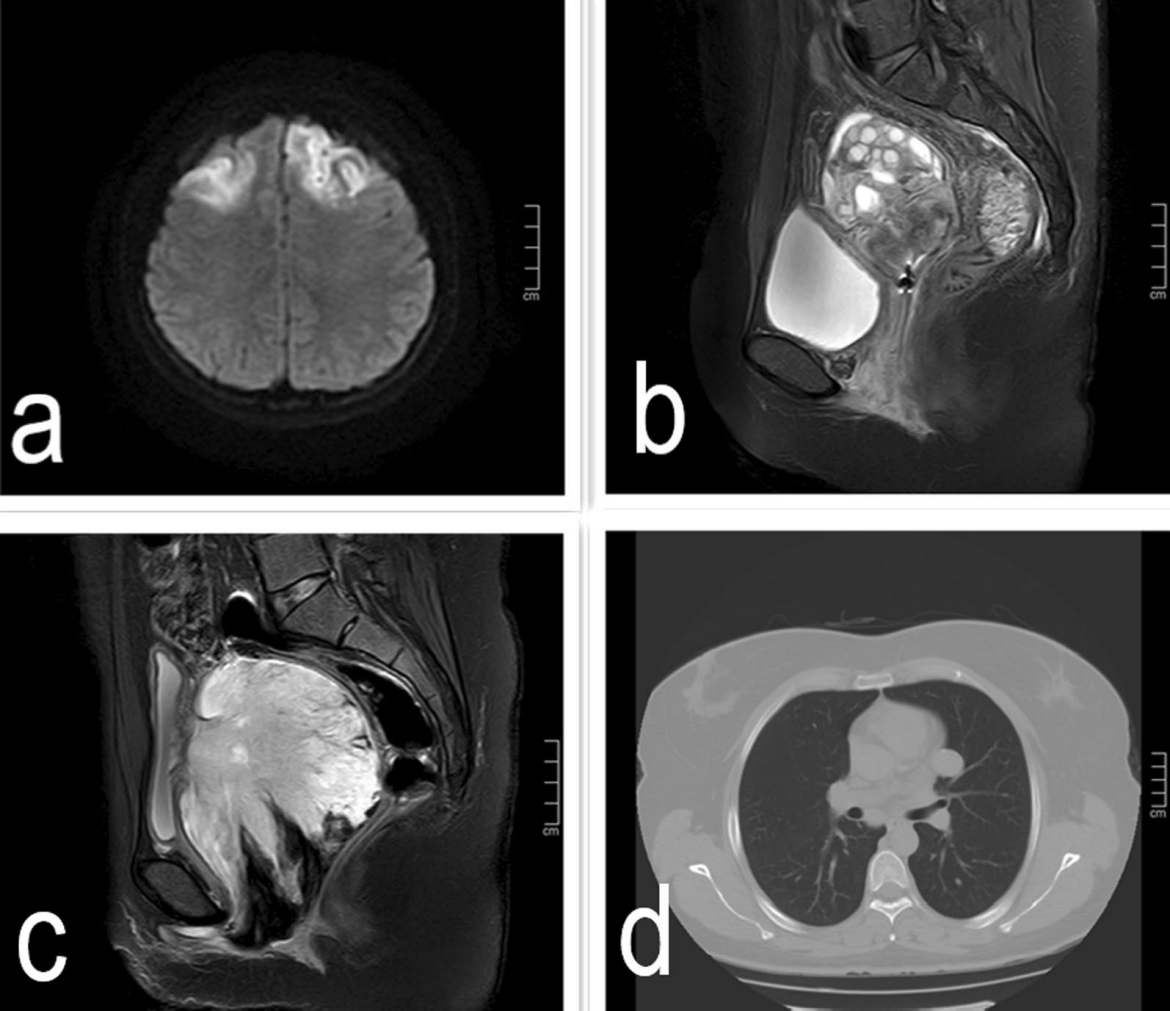
**a** Head CT scan: venous sinus thrombosis at admission; **b** pelvic MRI scan: pelvic recurrence two and a half months post hysterectomy: recurrent tumour; **c** pelvic MRI scan: pelvic recurrence five months post hysterectomy; **d** lung CT scan: lung metastasis five months post hysterectomy

**Fig. 2:**
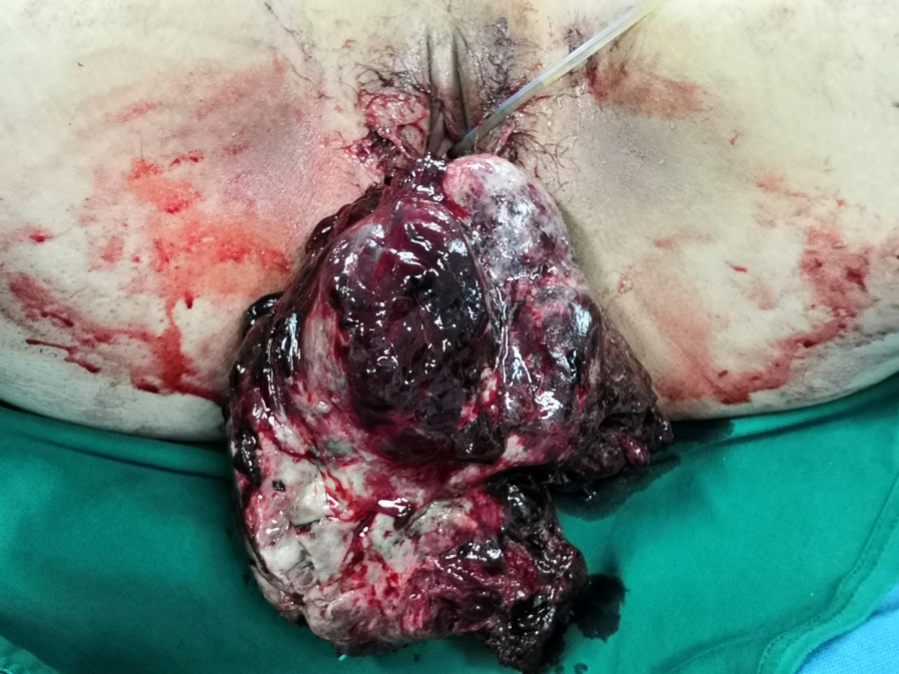
Complete uterine inversion secondary to uterine RMS

**Fig. 3:**
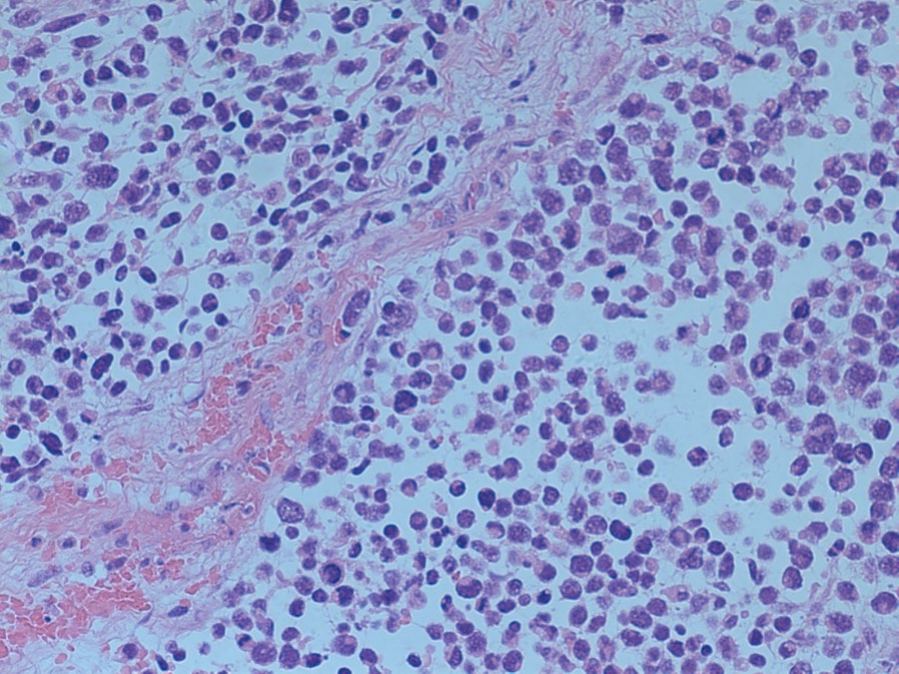
Histology demonstrating embryonal rhabdomyosarcoma with relatively uniform spindle cells with dark nuclei and inconspicuous nucleoli (HE stain, 20×)

A 19-year-old nulliparous Chinese woman presented to the emergency room of our institution under sedation due to unconsciousness and convulsions. Her body mass index was 34.5. Her medical history was notable for a 6-month history of abnormal uterine bleeding (AUB) that persisted despite oral contraceptives use. She had no history of exposure to the AstraZeneca COVID-19 vaccine or other high-risk drugs for thrombosis. Two days prior, she was admitted to a local hospital with severe headaches, nausea, vomiting, delayed reactions, expressive aphasia, and seizures, and was diagnosed with CVT. Intravenous administration of heparin and symptomatic treatment were administered, which were ineffective. The patient continued to have seizures. Then she was transferred to our institution. A computerised tomography (CT) scan of the head demonstrated venous sinus thrombosis (Fig. 1a). Emergency cerebrovascular angiography with femoral artery and femoral vein intubation was performed. The patient received thrombolytic therapy via femoral intravenous urokinase on the same day that she presented to our institution and this was continued for 7 days. Subsequently, her general condition improved. On the 9th day of hospitalisation, the patient developed vaginal bleeding. Considering this is a common complication of thrombolytic therapy, physicians did not treat the vaginal bleeding. On the 11th day of hospitalisation, she developed massive vaginal bleeding and inspection revealed a mass protruding from the vagina. Her haemoglobin level dropped from 10.8 g/dL to 6.3 g/dL in 3 h, and a gynaecology review was requested. Physical examination revealed pallor of the skin and mucous membranes, with no signs of visceromegaly, ascites or palpable masses on abdominal examination. Gynaecologic examination revealed that the vaginal mass was approximately 17 × 15 × 10 cm, and the tissue showed local congestion, infection, and necrosis (Fig. 2). Digital vaginal examination revealed that the mass was occupied the entire vaginal cavity with a thick pedicle, and the cervix was not visible or palpable. Rectal examination revealed no tumour effects. A cervical tumour was suspected. After a brief discussion of all therapeutic options within our clinical team, and given the patient’s poor general condition and acute cerebral thrombosis,emergency transvaginal resection of the mass was performed to achieve haemostasis. The mass was removed completely from the pedicle. Postoperative pathology suggested rhabdomyosarcoma of the embryonal type (Fig. 3). The tumour pedicle was negative. Immunohistochemical results showed the following staining patterns: CD10(+), CD117(−), CD34(−), CD68(+), pan-CK(−), desmin(+), DOG1(−), Ki-67(+), LAC(−), lysozyme(−), MyoD1(+), myogenin(+), S-100(−), SAM(−), and vimentin(+). The patient was misdiagnosed with rhabdomyosarcoma of the cervix. On the 15th day after the mass resection, laparoscopy and hysterectomy were planned. Intraoperative exploration showed no uterus in the pelvic cavity. The bilateral adnexa near the uterus adhered to the scar on the pelvic floor at the vaginal vault. The visible parts of the bilateral fallopian tubes and ovaries were grossly normal. There was no palpable pelvic or para-aortic lymphadenopathy. Based on the pathology results, we believed that the mass that was removed vaginally was a completely inverted uterus. Finally, we performed laparoscopic resection of the bilateral fallopian tubes and residual cervical tissue. Postoperative pathology was negative for tumours. Finally, she was diagnosed with uterine rhabdomyosarcoma complicated by CVT and uterine inversion. Chemotherapy with radiation was recommended; however, the patient asked for adjunctive treatment to be postponed and she began her first cycle of chemotherapy 43 days after the first surgery. The patient was treated with chemotherapy consisting of vincristine, actinomycin D and cyclophosphamide (VAC). Seventy-two days after the first surgery, she developed recurrence of a 5 cm mass in the pelvis (Fig. 1b).The mass continued to grow despite further chemotherapy cycles, and during her fourth cycle of chemotherapy, pelvic magnetic resonance imaging (MRI) revealed a rapid increase in the size of the mass to 16 cm (Fig. 1c), and a chest CT scan revealed multiple metastatic nodules in both lungs(Fig. 1d). The patient continued to experience vaginal bleeding and received multiple blood transfusions. The tumor occupied the pelvic cavity and caused urethral obstruction. As the patient experienced tumour progression despite chemotherapy, she was started on palliative treatment and died in less than 6 months.

## Discussion

Primary RMS of the uterus is an exceedingly rare and under-recognized aggressive neoplasm [3]. The signs and symptoms of uterine RMS typically include AUB, pelvic pressure/pain, and/or a uterine mass, although some women are asymptomatic [4]. Cases of bleeding accompanied by foul-smelling vaginal discharge have also been reported [5]. In this case report, the patient presented with symptoms of CVT and her medical history was notable for a 6-month AUB, which was uncontrolled with oral contraceptives. The symptoms of AUB were incompletely investigated; this was partly because she was obese, virgo intacta, and had irregular periods. AUB is a common condition, especially in adolescents, and malignant tumours of the uterus are rare causes of AUB. However, patients with persistent AUB in the absence of a clear cause should be carefully investigated to exclude malignant tumours of the uterus and avoid delays in diagnosis and treatment. Patients with persistent AUB should undergo imaging, pelvic examination and hysteroscopy with biopsy, if indicated.

In this case report, the patient presented to the emergency department under sedation due to CVT. CVT is a rare but potentially devastating type of stroke that tends to occur in young adults, especially women [6]. It is usually associated with a transient prothrombotic state, such as pregnancy, puerperium, and exposure to oral contraceptives [7]. It is rare in patients with cancer. To our knowledge, this is the first case of uterine rhabdomyosarcoma complicated with CVT. Malignancy is associated with an increased risk of venous thromboembolism (VTE) [8] due to multiple factors including malignancy per se and disease- and treatment-related complications [9]. Patients with malignancy also experience higher rates of VTE recurrence and bleeding complications during VTE treatment [10]. The use of anticoagulants can increase the risk of emergency surgery and cause hesitancy among surgeons to operate. This was also the case in our patient who had bleeding and thrombosis, and therefore conservative surgery was chosen as the safest option. In addition, systemic chemotherapy causes thrombocytopenia, which increases the risk of bleeding. She experienced multiple vaginal bleeding requiring transfusion during adjuvant therapy. Moreover, VTE is recognised as a manifestation of aggressive tumours. Previous studies have demonstrated that women who develop VTE have worse survival outcomes [11, 12]. In this case report, CVT was secondary to uterine RMS. Currently, uterine RMS remains an underrecognized aggressive neoplasm and the optimal treatment has not been established. Uterine RMS is treated with a multidisciplinary approach including surgery, chemotherapy, and radiotherapy to improve the cure rate and prolong survival time. Data regarding adjuvant therapy for uterine RMS are limited. The Intergroup Rhabdomyosarcoma study group compared the various modes of therapy in RMS and concluded that chemotherapy comprising VAC is the gold standard treatment option with or without radiotherapy for children and adolescents. Although radiotherapy reduces the frequency of recurrence, its impact on 5-year survival has not been proven [13]. Patients with uterine RMS have a poor prognosis, including our patient who experienced a rapid spread of neoplastic metastases to the lungs and died within 6 months. Further research is required to to explore more effective and new treatments.

The second complication in this case was a non-puerperal uterine inversion. Non-puerperal uterine inversion is an uncommon complication of a malignant uterine mass. It is usually precipitated by tumours exerting traction force on the fundus of the uterus, turning the uterus partially or completely inside out. Data regarding the treatment of uterine RMS associated with inversion are limited. A review of the English literature identified only 6 cases of uterine inversion due to RMS from 1887 to date, including the present case. Interestingly, those 6 patients ranged in age from 15 to 22 years (mean 18.5). All 6 tumours were large, and the average tumour size was 8.8 cm. Hysterectomy was the surgical treatment of choice in all cases. Addition details are provided in Table 1. Regarding uterine inversion due to malignancy, the first surgical manoeuvre is to return the uterus to its normal anatomic position, followed by abdominal hysterectomy [14]. However, this type of surgery described in the literature may not be appropriate for patients such as ours, as the inversed uterus completely prolapsed of the vagina, and thus it was too big to return to its normal anatomic position. Moreover, returning the massive inverted uterus to its normal anatomic position through the vagina is not preferable because the tumour will be squeezed during this reduction process. It will increase the risk of tumour seeding. For cases like ours, transvaginal excision of the completely inversed uterus may be a potential surgical option. However, as the enlarged and inversed uterus was removed vaginally, greater difficulties were involved than would have been encountered with transabdominal hysterectomy.

**Table 1:** Summary of the literature

References	Age (years)	Histology	presentation	Mass size (cm)	Treatment	Follow-up ststus (mo)
Case et al. [15]	21	ARMS	Bleeding	10.2 × 8.2	NACT, TAH + BSO,CT	ANED(20)
Ojiwang et al. [16]	16	ERMS	Bleeding	10.5 × 9.0	NACT,TAH, RT	
Sharma et al. [17]	18	RMS	Bleeding vaginal mass	17 × 15	TAH + BSO,CT	Pelvic recurrence(12)
da Silve et al. [18]	15	ERMS	Bleeding vaginal mass	11 × 9.7	TAH,CT,RT	Vaginal recurrence(1.5) DOD(9)
Ambreen et al. [19]	22	ERMS	Bleeding vaginal mass	10 × 8	TAH + BSO, CT,RT	Live metastasis(12) DOD(20)
Current Case	19	ERMS	Venous sinus thrombosis	18 × 16	VH + BSO,CT	Pelvic recurrence(2.4) DOD(6)

ARMS, alveolar RMS; ERMS, embryonal RMS; TAH, total abdominal hysterectomy; BSO, bilateral salpingo-oophorectomy; VH, vaginal hysterectomy; CT, chemotherapy; RT, radiotherapy; NACT, neoadjuvant chemotherapy; ANED, alive with no evidence of disease; DOD, dead of disease;

## Conclusions

In this article, we present a case of uterine rhabdomyosarcoma complicated by cerebral venous thrombosis and uterine inversion in a young woman. The patient had a history of her abnormal uterine bleeding which was not carefully investigated, leading to a delay in the diagnosis of uterine rhabdomyosarcoma. This case demonstrates the importance of ruling out uterine neoplasms in young women presenting with persistent AUB. In addition, careful anatomic evaluation should be performed in cases of any vaginal masses before proceeding with surgical intervention to ensure that the correct procedure is performed.

## Data Availability

The datasets used and analyzed during the current study are available from the corresponding author on reasonable request.

## Abbreviations

RMS: Rhabdomyosarcoma
CVT: Cerebral venous thrombosis
AUB: Abnormal uterine bleeding
VAC: Vincristine, actinomycin D and cyclophosphamide
